# Fetal cardiac MRI

**DOI:** 10.1186/1532-429X-17-S1-P220

**Published:** 2015-02-03

**Authors:** Ming Zhu

**Affiliations:** Radiology Dept, Shanghai Children’s Medical Center, Shanghai, China

## Background

MRI has been confirmed as an important tool for fetal central nervous system and other diseases. MRI has also emerged as a powerful approach to imaging heart diseases in infants. But fetus with heart diseases diagnosed by MRI has relative rarely been reported. Now most doctors agree that fetal cardiac MRI can provide some additional information when the patient was with some special conditions such as obesity, uterine myoma, twins and oligohydramnios. There also has been general agreement that fetal cardiac MRI can provide some additional information for fetal cardiac tumor, heart diverticulum, pericardium cyst, pericardium effusion and others. The question is if the patient was without any special conditions such as obesity and oligohydramnios, the fetal cardiac MRI still can or can not provide enough information for fetal congenital heart disease (CHD) which is more difficult to diagnose.

## Methods

We review our 280 cases of fetal CHD confirmed by postnatal imaging examination. MRI was performed using two 1.5T unit(Signa Echospeed; GE and Achieva Nova dual; Philips). Among the 280 cases, fetal cardiac MRI was performed at 17 to 39 weeks' gestation (mean 27 weeks). Imaging sequences included 2D SSFP, non-gated SSFP Cine and SS-TSE sequence. 2D SSFP is the most important sequences. B-TFE sequences (Philips), FOV: 280-310 mm, TR/TE: shortest/ shortest; flip angle: 70-85°, Overlap slices, section thickness, 4~7 mm; spacing, -2~-5 mm, matrix, 224×224.

## Results

The 280 cases of CHD included VSD, AVC, CoA, IAA, double aortic arch, LSVC,TOF, D-TGA, L-TGA, PA/VSD, PA/IVS, DORV, hypoplastic left ventricle syndrome, TA, SV and others. MRI provided additional important information to fetal echocardiography at 51(18%) cases. Four chamber view still is the most important view. The fetal MR imaging of the transverse view of aortic arch just like the "three vessels view" in fetal echo also is very important. A lot of congenital cardiovascular anomalies can be found some clues at the two views.

## Conclusions

Fetal echo still was the first choice method in fetal CHD. Now MRI can become an alternative imaging modality for fetal CHD. When compared to echocardiography, fetal MRI have some limitations. MRI sometimes missed small VSD and mild valvular stenosis. We never think someday MR can instead echo. But we believe MRI really can provide some additional important information in fetal CHD. SSFP overlap slices scan, the radiologist who read fetal cardiac MRI understanding enough about fetal CHD and understanding enough about how to read fetal imaging are the key points.

